# Preparation and Characterization of a New Polymeric Multi-Layered Material Based K-Carrageenan and Alginate for Efficient Bio-Sorption of Methylene Blue Dye

**DOI:** 10.3390/polym13030411

**Published:** 2021-01-28

**Authors:** Chiraz Ammar, Fahad M. Alminderej, Yassine EL-Ghoul, Mahjoub Jabli, Md. Shafiquzzaman

**Affiliations:** 1 Department of Fashion Design, College of Design, Qassim University, Al Fayziyyah Buraidah 52383, Saudi Arabia; c.ammar@qu.edu.sa; 2 Textile Engineering Laboratory, University of Monastir, Monastir 5019, Tunisia; 3 Department of Chemistry, College of Science, Qassim University, Buraidah 51452, Saudi Arabia; f.alminderej@qu.edu.sa; 4 Department of Chemistry, College of Science Al-Zulfi, Majmaah University, Zulfi 11932, Saudi Arabia; m.jabli@mu.edu.sa; 5 Textile Materials and Processes Research Unit, Tunisia National Engineering School of Monastir, University of Monastir, Monastir 5019, Tunisia; 6 Department of Civil Engineering, College of Engineering, Qassim University, Buraidah 51452, Saudi Arabia; shafiq@qec.edu.sa

**Keywords:** polyelectrolyte multi-layers, sodium alginate, k-carrageenan, cellulosic nonwoven textile, surface functionalization, characterization, bio-sorption, isotherms

## Abstract

The current study highlights a novel bio-sorbent design based on polyelectrolyte multi-layers (PEM) biopolymeric material. First layer was composed of sodium alginate and the second was constituted of citric acid and k-carrageenan. The PEM system was crosslinked to non-woven cellulosic textile material. Resulting materials were characterized using FT-IR, SEM, and thermal analysis (TGA and DTA). FT-IR analysis confirmed chemical interconnection of PEM bio-sorbent system. SEM features indicated that the microspaces between fibers were filled with layers of functionalizing polymers. PEM exhibited higher surface roughness compared to virgin sample. This modification of the surface morphology confirmed the stability and the effectiveness of the grafting method. Virgin cellulosic sample decomposed at 370 °C. However, PEM samples decomposed at 250 °C and 370 °C, which were attributed to the thermal decomposition of crosslinked sodium alginate and k-carrageenan and cellulose, respectively. The bio-sorbent performances were evaluated under different experimental conditions including pH, time, temperature, and initial dye concentration. The maximum adsorbed amounts of methylene blue are 124.4 mg/g and 522.4 mg/g for the untreated and grafted materials, respectively. The improvement in dye sorption evidenced the grafting of carboxylate and sulfonate groups onto cellulose surface. Adsorption process complied well with pseudo-first-order and Langmuir equations.

## 1. Introduction

The discharge of dyes from industries and their elimination has received much attention as they can damage human health and animals [1,2,3]. Indeed, dye molecules are resistant to natural degradation, allergenic, carcinogenic, and stable in the presence of oxidizing agents [4,5,6]. This interesting topic requires the development of various technologies to treat colored waters. Biological treatment and coagulation/flocculation processes are viewed as ineffective to treat soluble dyes [7,8,9]. Adsorption appeared more effective as it is simple and economic and it is especially used to remove pollutants, which are not easily biodegradable. Thus, a specific attention is devoted to explore new adsorbents, which could be cheaper, proficient, and easy regenerated.

In this sense, several polymeric adsorbents were used for the removal of dyes from contaminated matters [10,11,12]. Concerning textile adsorbent materials, they are rarely investigated in the literature. Some sorbent material based synthetic functionalized textiles were studied for the removal of cationic dyes. Despite their pretreatment and/or functionalization, adsorption capacities were limited due to their hydrophobicity [13,14,15]. Cellulose, alginates, and k-carrageenan biopolymers were naturally abundant polysaccharides. Cellulose is recognized for its good hydrophilicity [16,17,18,19,20,21]. Alginate, a polysaccharide biopolymer, is an essential component of the cell wall of brown algae. It could be an excellent bio-sorbent of organic dyes due to its high contents of carboxylic and hydroxyl functional groups [22,23,24,25]. K-carrageenan is a natural sulfated polysaccharide extracted from red edible seaweeds. It is widely used in the food industry, owing to gelling, thickening, and stabilizing properties [26,27]. As examples of works conducted in previous literatures, Rahman et al., in a comprehensive review, discussed synthesis, characteristics, and methylene blue adsorption of various cellulose nanocrystal-based hydrogels [28]. They indicated that the addition of other polysaccharides including chitosan and alginate into cellulose displayed remarkably improved adsorption capacities for methylene blue. Guesmi et al. demonstrated that the addition of sodium alginate (5–20%) to hydroxyapatite improved well methylene blue adsorption [29] and the adsorption capacity increased from 77.51 to 142.85 mg/g, using hydroxyapatite and hydroxyapatite-alginate, respectively. In our previous work, we have demonstrated that the adsorption of methylene blue onto extracted cellulose, from Aegagropila L., reached 109 mg/g and it was only about 47mg/g for the raw marine macroalgae [30]. Yang et al. synthesized gel beads based on k-carrageenan and graphene oxide [31] and observed that the adsorption capacity of the gel beads for methylene blue attained 658.4 mg/g at 25 °C. As globally observed, the developed materials-based polysaccharides are outstanding adsorbents with excellent adsorption capacities of cationic dyes.

In line with this emergent topic, the current study proposes a novel adsorbent design based on polyelectrolyte multi-layered (PEM) biopolymeric material as a potent bio-sorbent. This is formed by an alternation of layers of two polyelectrolyte biopolymers. The first layer is composed of alginate polyanion and the second is obtained after the reticulation of the citric acid with k-carrageenan polyanion polymer. The PEM system is crosslinked to cellulosic natural material and applied as adsorbent of cationic dyes. Indeed, the proposed technique offers the adsorption characteristics of not only cellulose as the main support but also k-carrageenan and sodium alginate as immobilized biopolymers. The system provides massive hydroxyl, sulfonate, and carboxylate groups suggesting therefore the use of the resulting material as a bio-sorbent of cationic species. Therefore, the importance of our proposal lies in the preparation of a new PEM biomaterial based on alginate and k-carrageenan crosslinked to cellulosic material, which is very rich in anionic functional groups. In addition, the investigation of the layer-by-layer functionalization method in the adsorption of textile rejects is a novel study and was not reported in the literature. The prepared materials were characterized using FT-IR, SEM, TGA, and DTA. The bio-sorbent performances were evaluated under different experimental conditions including pH, time, temperature, and the initial dye concentration. The theoretical kinetic and isotherms equations were used to analyze the experimental data.

## 2. Experimental

### 2.1. Materials

A non-woven cellulosic textile material was used in this study. Its surface weight was 240 g/m² and the thickness had an average of 0.6 mm. The textile is a 3-dimensional calendared fiber network. Sodium alginate (AG) was supplied from Sigma-Aldrich with a medium molecular weight (30,000 g/mol) having a degree of deacetylation of 70%. Citric acid (CTR, 226.2 g/mol) and Kappa carrageenan (a fine white powder) were purchased from Sigma-Aldrich. All chemicals were used without further purification. Methylene blue (MB) was supplied by Central Drug House (India).

### 2.2. Preparation of PEM Bio-Sorbent

PEM bio-sorbent was prepared via an alternating grafting process of sodium alginate and k-carrageenan using citric acid as a crosslinking agent. The process of grafting of the cellulosic material is based on a pad-dry technique. PEM bio-sorbent was carried out by functionalizing the cellulosic material with alternating baths in sodium alginate/citric acid solution and then in a K-carrageenan solution. The PEM is deposited by alternating successive baths according to the “layer-by-layer deposition” method. Different pairs of layers (from 1 to 8) are prepared for bio-sorbent cellulosic material. Each impregnation was performed in a total volume of 50 mL. All polymer solutions are completely renewed after the deposition of 2 pairs of layers. The samples (4 cm × 4 cm) were treated in a solution of soluble sodium alginate polymer (0.4% *w/v*) and citric acid (0.4% *w/v*) under constant stirring at 170 rpm for 20 min at room temperature. After each impregnation, the samples were dried 20 min at 90 °C. After drying, the samples were impregnated in a solution of k-carrageenan (at 0.8% *w/v*) with stirring (170 rpm) for 20 min at room temperature. The cycle was repeated as many times as necessary. At each end of the cycle, a pair of layers was thus deposited and crosslinked on the cellulose material.

During the preparation process of PEM materials, the weight gain was calculated after depositing each pair of layers of sodium alginate and k-carrageenan. The following formula was applied illustrating the weight gain as a function of the number of pairs of grafted copolymer layers.

(1) $$\%-Weight\;gain\;\left(n\right)=\;\frac{\left(m_{n}-m_{i}\right)}{m_{i}}\times \;100$$

where *n* is the number of pairs of layers and *m_i_* and *m_n_* are the sample weights before and after functionalization, respectively.

### 2.3. Characterizations

For the analysis of the chemical structure of prepared multilayered adsorbent, infrared spectroscopy analysis was conducted using a FT-IR spectrometer (Agilent Technologies, Cary 600 Series FTIR Spectrometer) via the attenuated total reflection mode (ATR). Spectra of cellulose material and multilayered bio-adsorbent polymeric system were recorded at a range of 4000 to 400 cm^−1^, and with a resolution of 2 cm^−1^.

Thermal stability and different thermal properties of natural cellulosic material and PEM bio-sorbent were determined by thermogravimetric measurements using TA Instruments apparatus. The fixed parameters for different analysis were a heating rate of 10 °C min^−1^ and a temperature from 25 to 600 °C.

Surface morphology was assessed using a scanning electron microscopy (FEI Quanta SEM). An accelerating voltage of 5 KV with various magnification essays was applied for the surface analyzing of different samples. The SEM analysis was preceded by a coating procedure of samples with a carbon layer to enhance their conductivity.

### 2.4. Adsorption Experiments

The adsorption tests were carried out in a batch reactor by stirring the colored synthetic solutions in the presence of each of the adsorbents at a constant agitation speed (150 rpm). We studied the effect of the main parameters influencing the adsorption capacity such as pH (ranged from 3 to 9), contact time (in a range of 0 to 120 min.), initial dye concentration (varied from 50 to 1000 mg/L), and temperature (22, 40, and 60 °C). The residual concentration of each of the dyes was determined using an UV/visible spectrophotometer (Shimadzu UV-2600).

The adsorbed methylene blue dye amount (q(mg/g)) onto the surface of PEM crosslinked to non-woven cellulosic textile material was calculated using the following formula:

(2) $$q\left(\mathrm{mg}/g\right)=\;\frac{\left(C_{0}-C_{e}\right)\cdot V}{m}$$

where *C*_0_ and *C_e_* are the initial and residual concentration (mg/L), respectively, *V* is the volume of methylene blue dye (L) used for the adsorption, and m is the mass of the biosorbent (g) used for the adsorption.

## 3. Results and Discussion

### 3.1. Preparation of PEM Biopolymer Adsorbent

**Figure 1:**
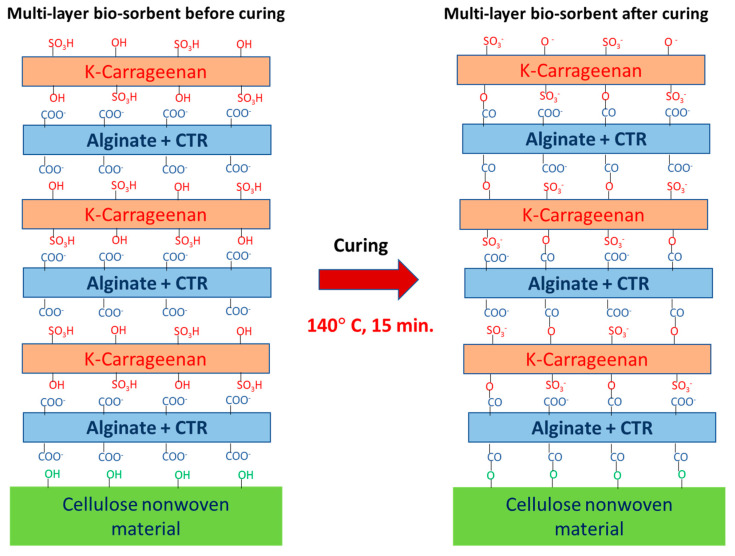
Illustration of the functionalization of cellulosic material upon polyesterification reactions via CTR (Citric acid) crosslinking, leading to the polyanionic PEM bio-sorbent system after curing at 140 °C for 15 min.

**Figure 2:**
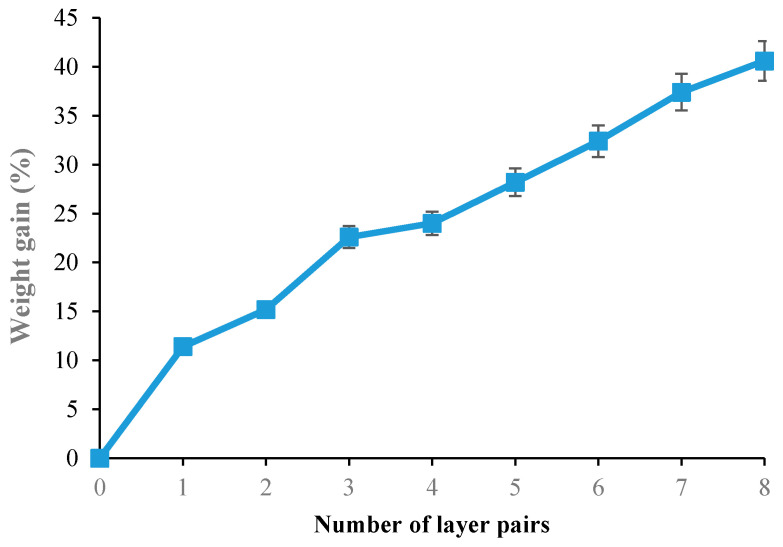
Weight gain variation as function of the number of layers grafted onto the cellulosic bio-sorbent material.

The design of PEM was carried out by grafting the cellulosic material with alternating layers of sodium alginate and K-carrageenan biopolymers crosslinked by citric acid (Figure 1). The layer-by-layer deposition technique was used to elaborate different multi-layered bio-sorbent (from 1 to 8-layer pairs). The variation of the weight gain according to the number of grafted layers of sodium alginate and K-carrageenan was presented in Figure 2. We noticed the progressive increase of total weight gain with the number of polymeric layers grafted to the cellulosic material. Results revealed a more significant increase of the weight gain within 3-pairs-layers finishing the cellulosic sample. For the characterization and adsorption investigations, the samples treated with three polymeric layers of reticulated alginate and K-carrageenan will be investigated.

### 3.2. FT-IR Spectroscopy Analysis

**Figure 3:**
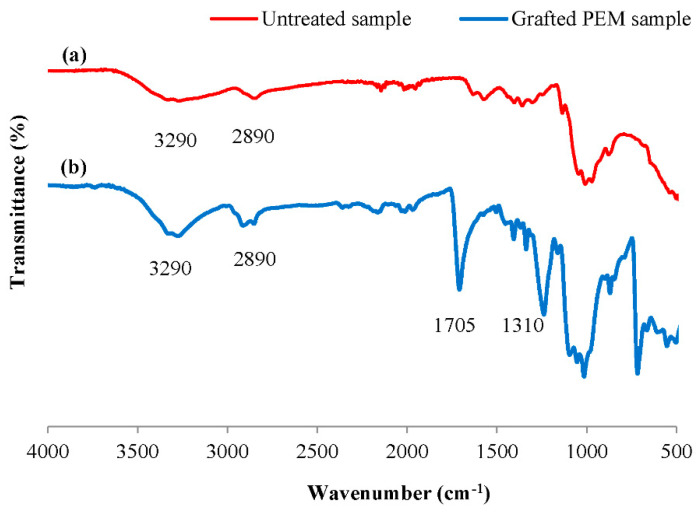
FT-IR-ATR spectra of virgin cellulosic sample (**a**) and PEM grafted bio-sorbent material (**b**).

FT-IR-ATR analysis was performed in order to characterize the chemical grafting and conception of the bio-sorbent material via the identification of the different functional groups appearing after functionalization. This characterization was applied to analyze both the untreated cellulosic sample and the PEM grafted adsorbent material. Spectra of untreated cellulosic sample and grafted PEM material (3-layers PEM) were showed in Figure 3. Different new peaks appeared in the PEM grafted cellulosic material proving the chemical grafting. A peak centered at 1705 cm^–1^ appeared referring to the ester groups confirming the polyesterification reaction established between carboxylic groups of citric acid and hydroxyl functions of both alginate and carrageenan biopolymers. This is in line with previous research studies, in which FT-IR were used to confirm the evidence of a polyesterification reaction between cellulosic material and different polysaccharides via polycarboxylic acids as crosslinking agents [32,33,34]. A peak closed to 1310 cm^–1^ appeared clearly, which corresponded to the symmetric stretching vibration of the carboxylic acid groups COO^_^ of galacturonic acid of alginate grafted polymer [35]. In addition, a more significant wide peak around to 3290 cm^–1^ appeared with grafted PEM material was attributed to hydroxyl groups of cellulose, alginate, and carrageenan grafted polymers. Briefly, FT-IR analysis of the two spectra, permitted us to confirm the chemical interconnection of the PEM bio-sorbent system and indicated the efficiency of the applied grafting chemical process.

### 3.3. TGA and DTA Analysis

**Figure 4:**
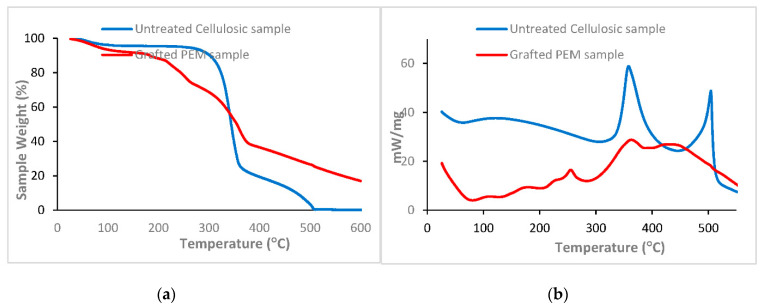
TGA (**a**) and DTA (**b**) thermograms of virgin cellulosic sample and grafted PEM bio-sorbent material.

Figure 4 showed thermograms of untreated and grafted PEM samples. The untreated cellulosic sample showed only one zone showing one temperature of degradation referring to the cellulosic material closed to 370 °C. However, two distinct parts appeared in the thermogram of the designed PEM sample. The first with a temperature of degradation around 250 °C was attributed to the crosslinked polymer of alginate and K-carrageenan. The second closed to 370 °C, with a high loss of weight referring to the degradation of the cellulosic sample. The observed weight loss for the two samples, around 100 °C, was due the evaporation of water absorbed inside their structures. At this temperature we noticed a higher weight loss in the case of the grafted PEM material. This was due to the increase of the hydrophilicity after biopolymers grafting onto the cellulosic samples. Furthermore, the prepared PEM samples presented a higher residual weight after degradation (20%) compared to the untreated one (0%). This proved well the improvement thermal stability of the PEM bio-sorbent material after grafting.

DTA results confirmed the TGA analysis and revealed one significant peak in the derivative signal ascribed to the cellulose degradation in the case of untreated sample. For the untreated PEM sample two principal peaks appeared, which were attributed to the degradation of the grafted polymers (at 250 °C) and the degradation of the cellulosic material (at 370 °C).

### 3.4. Morphological Analysis

**Figure 5:**
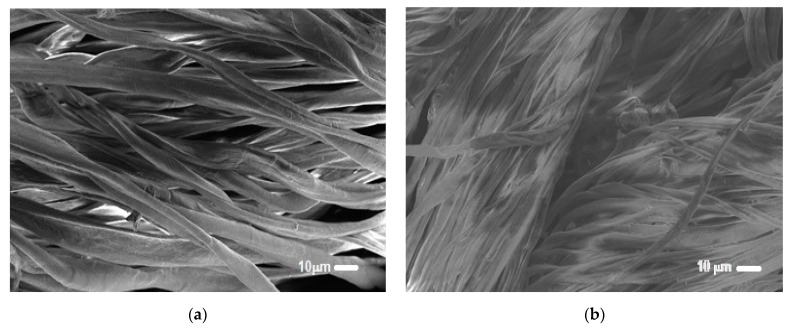
SEM micrographs of (**a**) untreated cellulosic sample and (**b**) PEM grafted bio-sorbent material.

Figure 5 showed the micrographs of untreated cellulosic sample and grafted PEM material with 3-layer pairs. We noticed a significant surface modification after grafting. The microspaces between fibers were filled with the layers of the two functionalizing polymers. The grafted PEM revealed a higher surface roughness compared to the virgin sample. This modification of the surface morphology confirmed the stability and the effectiveness of the applied method of grafting.

### 3.5. Application to the Adsorption of Methylene Blue

**Figure 6:**
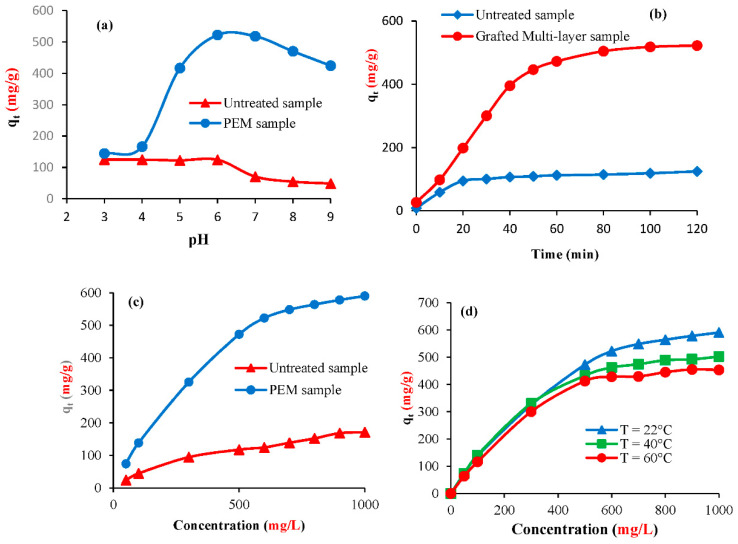
(**a**) Effect of pH on methylene blue adsorption (C_0_ = 600 mg/L, t = 120 min), (**b**) effect of time, (**c**) effect of initial dye concentration, and (**d**) effect of temperature.

In this investigation, the prepared untreated and grafted materials were applied as adsorbents of methylene blue in synthetic medium by varying pH, time, initial dye concentration, and temperature. Figure 6a represents the evolution of the adsorbed methylene blue amount as a function of pH. The maximum adsorbed amount was reached at pH = 6. In fact, under high acidic conditions, the positively charged methylene blue ions opposed the positively charged adsorbent surface leading to low adsorbed rates. At higher pH values (pH < 6), the adsorbent surface became negative, favoring an electrostatic interaction with methylene blue. At basic conditions, the adsorbed dye amount decreases, which could be explained by the repulsion forces between dye molecules and biosorbent surface.

The time required to achieve equilibrium was observed at 120 min (Figure 6b). It is seen that the adsorption was fast during the first period of times (from 0 to 50 min) where more than 80% of target was accomplished. This trend could be explained by the fact that many adsorption sites are available during this first stage at the surface of the adsorbents. After this period of times, the adsorption attained a steady state, which could be interpreted by the saturation of the adsorption sites. Results showed also that the maximum adsorbed amount of methylene blue are 124.4 mg/g and 522.4 mg/g for the untreated materials and grafted ones, respectively. This difference in the sorption capacities could be explained by the addition of new functional groups (carboxylate and sulfonate groups) grafted on the surface of cellulose.

**Scheme 1:**
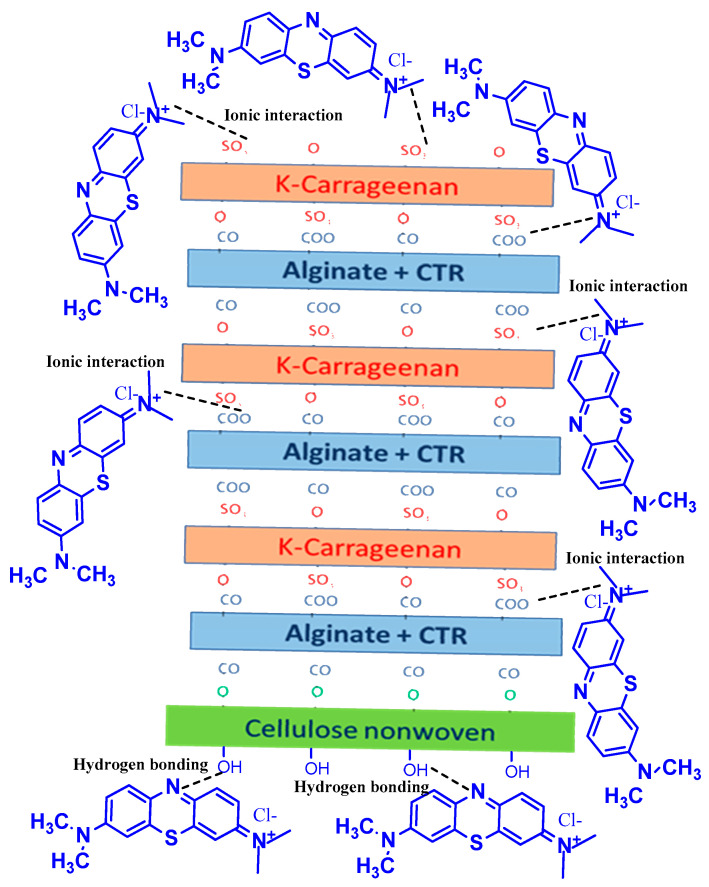
A schematic representation of hydrogen bonding and ionic interaction between methylene blue molecule and the surface of PEM crosslinked to non-woven cellulosic textile material.

Scheme 1 provides a schematic representation of hydrogen bonding and ionic interaction between methylene blue molecule and PEM crosslinked to non-woven cellulosic textile material surface. Indeed, the free hydroxyl groups of cellulose non-woven could interact with nitrogen atom of methylene blue via hydrogen bonding. However, the sulfonate groups (SO_3_^−^) of K-carrageenan and/or carboxylate groups of both sodium alginate (COO^−^) and CTR crosslinking agent could react with positive nitrogen atom (N^+^) through ionic interaction.

The adsorbed dye amount was found to greatly increase with the increase of initial methylene blue concentration. At equilibrium, it reached 171.4 mg/g for the untreated samples and 590.5 mg/g for the grafted samples (Figure 6c). This adsorbed amount depends on the temperature value and it decreased for example in the case of grafted samples from 590.5 mg/g at 22 °C to 453.5 mg/g at 60 °C (Figure 6d). This indicated that the adsorption of methylene blue was exothermic in this case. Indeed, at higher temperatures values, the dye could be desorbed from the samples.

### 3.6. Kinetic Modeling

**Figure 7:**
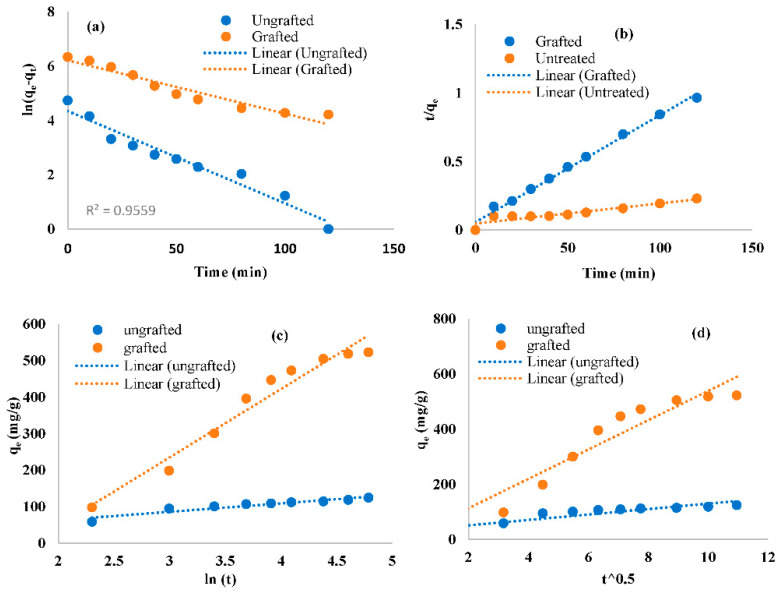
Kinetic data modeled using: (**a**) pseudo first order, (**b**) pseudo second order, (**c**) Elovich, and (**d**) intraparticular diffusion.

Kinetics data were significant to recognize the attraction between adsorbates and adsorbents at equilibrium state. It could suggest if the studied mechanism is physical and/or chemical, and mass transport. Herein, modeling kinetic data was assessed using pseudo first order, pseudo second order, Elovich, and intradiffusion kinetic models. Results are depicted in Figure 7. The computed kinetic parameters for the different equations were summarized in Table 1. The obtained curves and computed kinetic parameters indicated that pseudo-first-order equation could describe well the kinetic data (0.96 < R^2^). The correlation coefficients obtained within the pseudo-second-order were also high (0.95 < R^2^). These results indicated that the adsorption process is so complex and could be considered as physical and chemical [36]. The divergence of the plots for the intraparticle diffusion model from the origin suggests that this model was not the sole rate-controlling step [37].

### 3.7. Isotherms Investigation

**Figure 8:**
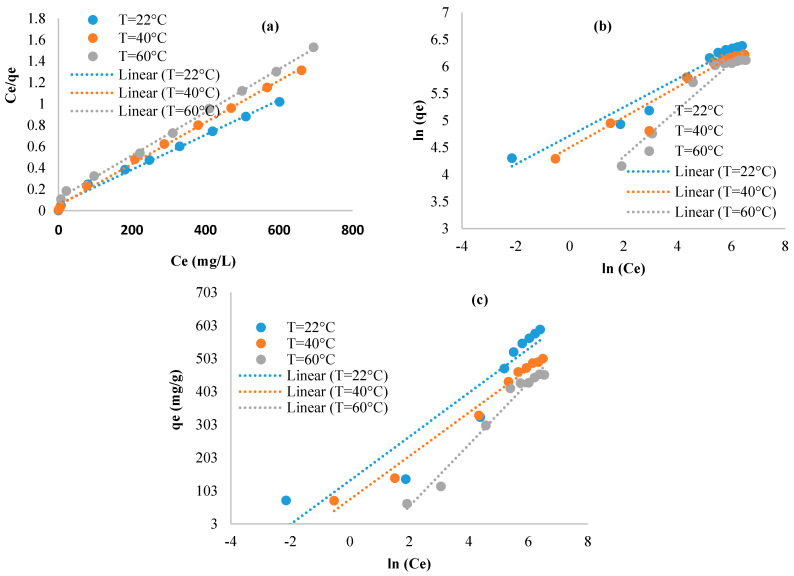
Isotherms data modeled using: (**a**) Langmuir, (**b**) Freundlich, and (**c**) Temkin.

The relationship between the grafted materials and the studied adsorbate was evaluated using the equations of Langmuir, Freundlich, and Temkin (Figure 8). The calculated parameters were shown in Table 2. We observed that the Langmuir equation fitted well with the experimental data (0.99 ≤ R^2^) compared to the other studied equations. This trend suggested that the adsorption process is a monolayer, and the sorption sites are homogeneous having similar adsorption capacities [38]. The values of (1/*n*) revealed adsorption intensity or surface heterogeneity. Indeed, when the value of 1/n ranges from 0.1 to 1.0, it therefore reflects a good adsorption [39]. In our study 2.3 ≤ *n* ≤ 3.8, which indicates that the adsorption of the molecules of methylene blue onto the surface of the grafted samples is so favorable. The decrease of the values of the adsorption energy constant (b_T_), determined from the Temkin model, with the increase of temperature indicated the exothermic nature of the adsorption mechanism. This is consistent with the trends observed within the effect of temperature discussed above.

## 4. Conclusions

In this study, a new multilayered polymeric bio-sorbent was elaborated using the layer-by-layer grafting method. The prepared cellulosic material based k-carrageenan and alginate crosslinked biopolymers were then chemically and morphologically characterized. Chemical grafting via polyesterification reactions and the stability of the multilayer functionalization were confirmed by FT-IR, TGA-DTA, and SEM analysis. Methylene blue dye was used as an adsorbate for the bio-sorption evaluation on the PEM grafting material in batch experiments. The influence of the different process parameters was studied with respect to the sorption equilibrium. The designed bio-sorbent showed excellent sorption capacities of MB with a capacity above 522.4 mg/g. The improvement in dye sorption was evidently due to the presence of many carboxylate and sulfonate groups of the crosslinked k-carrageenan and alginates grafted on the cellulosic surface material. The correlation of the experimental data with the theoretical equations showed that the kinetic data could be described with both pseudo first order and pseudo second order equations suggesting that the adsorption process involved physical and chemical interactions. The adsorption phenomenon occurred in heterogeneous adsorption sites and it was exothermic and spontaneous. Langmuir isotherm fitted better to adsorption data, indicating that the adsorption process was localized on a monolayer, and all adsorption sites on the adsorbent were homogeneous and had the same adsorption capacity. In brief, we developed a simple and new polymeric material for removing one of the most dangerous contaminants from textile industrial aqueous discharges. Further investigation could be extended for the exploration of this material in the removal of other pollutants such as metals and pesticides.

## Figures and Tables

**Table 1:** Summarized kinetic parameters for the grafted and ungrafted samples.

Pseudo First Order			Pseudo Second Order			Elovich			Intraparticle Diffusion		
Parameters	Ungrafted	Grafted		Ungrafted	Grafted		Ungrafted	Grafted		Ungrafted	Grafted
q_e_ (mg/g)	121	530	q_e_ (mg/g)	127.982	954.645	β (mg/g/min)	0.043	0.005			
k_1_ (1/min)	0.035	0.040	k_2_	1.07 × 10^−3^	1.5× 10^−5^	α (mg/g^/^min)	47.903	32.722	K	13.808	54.074
R^2^	0.967	0.992	R^2^	0.973	0.95	R^2^	0.9	0.958	R^2^	0.675	0.9217

Where: α (mg/g/ min) and β (mg/g/min) are Elovich constants; R^2^ is the regression coefficient.

**Table 2:** Summarized Langmuir, Freundlich, and Temkin parameters for the grafted samples.

Langmuir				Freundlich				Temkin			
	T = 22 °C	T = 40 °C	T = 60 °C		T = 22 °C	T = 40 °C	T = 60 °C		T = 22 °C	T = 40 °C	T = 60 °C
q_max_ (mg/g)	614.28	512.15	494.18	K_f_ (L/g)	112.1	90.3	31.8	b_T_ (J/mol)	36.98	39.69	29.66
K_L_ (L/g)	0.028	0.042	0.02	n	3.80	3.59	2.30	A_t_ (L/g)	7.55	3.24	0.25
R^2^	0.99	0.99	0.99	R^2^	0.9714	0.99	0.95	R^2^	0.87	0.98	0.98

## Data Availability

The data presented in this study are available on request from the corresponding author.

## References

[1] Venkat S.M., Vijay B.P.V. (2013). Kinetic and equilibrium studies on the removal of Congo red from aqueous solution using Eucalyptus wood (Eucalyptus globulus) saw dust. J. Taiwan Inst. Chem. Eng..

[2] Enamul H., Jong W.J., Sung H.J. (2011). Adsorptive removal of methyl orange and methylene blue from aqueous solution with a metal-organic framework material, iron terephthalate (MOF-235). J. Hazard. Mater..

[3] Karagozoglu B., Tasdemir M., Demirbas E., Kobya M. (2007). The adsorption of basic dye (Astrazon Blue FGRL) from aqueous solutions onto sepiolite, fly ash and apricot shell activated carbon: Kinetic and equilibrium studies. J. Hazard. Mater..

[4] Mahmoud D.K., Salleh M.A.M., Karim W.A., Idris A., Abidin Z.Z. (2012). Batch adsorption of basic dye using acid treated kenaf fibre char: Equilibrium, kinetic and thermodynamic studies. Chem. Eng. J..

[5] Fan L., Luo C., Li X., Lu F., Qiu H., Sun M. (2012). Fabrication of novel magnetic chitosan grafted with graphene oxide to enhance adsorption properties for methyl blue. J. Hazard. Mater..

[6] Asgher M., Bhatti H.N. (2012). Evaluation of thermodynamics and effect of chemical treatments on sorption potential of Citrus waste biomass for removal of anionic dyes from aqueous solutions. Ecol. Eng..

[7] Pagga U., Brown D. (1986). The degradation of dyestuffs: Part II Behaviour of dyestuffs in aerobic biodegradation tests. Chemosphere.

[8] Ishak S.A., Murshed M.F., Md Akil H., Ismail N., Md Rasib S.Z., Al-Gheethi A.A.S. (2020). The Application of Modified Natural Polymers in Toxicant Dye Compounds Wastewater: A Review. Water.

[9] Bahrpaima K., Fatehi P. (2019). Preparation and Coagulation Performance of Carboxypropylated and Carboxypentylated Lignosulfonates for Dye Removal. Biomolecules.

[10] Jabli M., Gamha E., Sebeia N., Hamdaoui M. (2017). Almond shell waste (Prunus dulcis): Functionalization with [dimethy-diallyl-ammonium-chloride-diallylamin-co-polymer] and chitosan polymer and its investigation in dye adsorption. J. Mol. Liq..

[11] Ali Khan M., Momina, Siddiqui M.R., Otero M., Alshareef S.A., Rafatullah M. (2020). Removal of Rhodamine B from Water Using a Solvent Impregnated Polymeric Dowex 5WX8 Resin: Statistical Optimization and Batch Adsorption Studies. Polymers.

[12] Sebeia N., Jabli M., Ghith A., Elghoul Y., Alminderej F.M. (2019). Populus tremula, Nerium oleander and Pergularia tomentosa seed fibers as sources of cellulose and lignin for the bio-sorption of methylene blue. Int. J. Biol. Macromol..

[13] Sharma M., Singh G., Vaish R. (2019). Diesel soot coated non-woven fabric for oil-water separation and adsorption applications. Sci. Rep..

[14] Ren Y., Guo J., Lu Q., Xu D., Qin J., Yan F. (2018). Polypropylene Nonwoven Fabric@Poly(ionic liquid)s for Switchable Oil/Water Separation, Dye Absorption, and Antibacterial Applications. ChemSusChem.

[15] Haji A., Mousavi Shoushtari A., Abdouss M. (2013). Plasma activation and acrylic acid grafting on polypropylene nonwoven surface for the removal of cationic dye from aqueous media. Desalination Water Treat..

[16] Salah F., El-Ghoul Y., Roudesli S. (2016). Bacteriological effects of functionalized cotton dressings. J. Text. Inst..

[17] Salah F., El-Ghoul Y., Alminderej F.M., Golli-Bennour E., Ouanes Z., Maciejak O., Jarroux N., Majdoub H., Sakli F. (2019). Development, characterization, and biological assessment of biocompatible cellulosic wound dressing grafted Aloe vera bioactive polysaccharide. Cellulose.

[18] El-Ghoul Y., Alminderej F.M. (2020). Bioactive and superabsorbent cellulosic dressing grafted alginate and Carthamus tinctorius polysaccharide extract for the treatment of chronic wounds. Text. Res. J..

[19] Salah F., El-Ghoul Y., Mahdhi A., Majdoub H., Jarroux N., Sakli F. (2017). Effect of the deacetylation degree on the antibacterial and antibiofilm activity of acemannan from Aloe vera. Ind. Crop. Prod..

[20] El-Ghoul Y., Salah F., Majdoub H., Sakli F. (2017). Synthesis and study of drug delivery system obtained via β-cyclodextrin functionalization of viscose/polyester dressings. J. Ind. Text..

[21] El-Ghoul Y., Ammar C., El-Achari A. (2014). New polymer based modified cyclodextrins grafted to textile fibers; characterization and application to cotton wound dressings. Int. J. Appl. Res. Text..

[22] Torres-Caban R., Vega-Olivencia C.A., Alamo-Nole L., Morales-Irizarry D., Roman-Velazquez F., Mina-Camilde N. (2019). Removal of Copper from Water by Adsorption with Calcium-Alginate/Spent-Coffee-Grounds Composite Beads. Materials.

[23] Siwek H., Bartkowiak A., Włodarczyk M. (2019). Adsorption of Phosphates from Aqueous Solutions on Alginate/Goethite Hydrogel Composite. Water.

[24] Zhang J., Deng R., Ren B., Yaseen M., Hursthouse A. (2020). Enhancing the Removal of Sb (III) from Water: A Fe3O4@HCO Composite Adsorbent Caged in Sodium Alginate Microbeads. Processes.

[25] Yang C.-H., Shih M.-C., Chiu H.-C., Huang K.-S. (2014). Magnetic Pycnoporus sanguineus-Loaded Alginate Composite Beads for Removing Dye from Aqueous Solutions. Molecules.

[26] Jancikova S., Dordevic D., Jamroz E., Behalova H., Tremlova B. (2020). Chemical and Physical Characteristics of Edible Films, Based on κ- and ι-Carrageenans with the Addition of Lapacho Tea Extract. Foods.

[27] Papoutsis K., Golding J.B., Vuong Q., Pristijono P., Stathopoulos C.E., Scarlett C.J., Bowyer M. (2018). Encapsulation of Citrus By-Product Extracts by Spray-Drying and Freeze-Drying Using Combinations of Maltodextrin with Soybean Protein and ι-Carrageenan. Foods.

[28] Rahman M.M., Rimu S.H. (2020). Recent development in cellulose nanocrystal-based hydrogel for decolouration of methylene blue from aqueous solution: A review. Int. J. Environ. Anal. Chem..

[29] Guesmi Y., Agougui H., Lafi R., Jabli M., Hafiane A. (2018). Synthesis of hydroxyapatite-sodium alginate via a co-precipitation technique for efficient adsorption of Methylene Blue dye. J. Mol. Liq..

[30] Sebeia N., Jabli M., Ghith A., Elghoul Y., Alminderej F.M. (2019). Production of cellulose from Aegagropila Linnaei macro-algae: Chemical modification, characterization and application for the bio-sorption of cationic and anionic dyes from water. Int. J. Biol. Macromol..

[31] Yang M., Liu X., Qi Y., Sun W., Men Y. (2017). Preparation of κ-carrageenan/graphene oxide gel beads and their efficient adsorption for methylene blue. J. Colloid Interface Sci..

[32] Alminderej M.F., El-Ghoul Y. (2019). Synthesis and study of a new biopolymer-based chitosan/hematoxylin grafted to cotton wound dressings. J. Appl. Polym. Sci..

[33] Uranga J., Nguyen B.T., Si T.T., Guerrero P., de la Caba K. (2020). The Effect of Cross-Linking with Citric Acid on the Properties of Agar/Fish Gelatin Films. Polymers.

[34] El-Ghoul Y. (2020). Biological and microbiological performance of new polymer-based chitosan and synthesized amino-cyclodextrin finished polypropylene abdominal wall prosthesis biomaterial. Text. Res. J..

[35] Zhao M., Yang N., Yang B., Jiang Y., Zhang G. (2007). Structural characterization of water-soluble polysaccharides from Opuntia monacantha cladodes in relation to their anti-glycated activities. Food Chem..

[36] Gucek A., Sener S., Bilgen S., Mazmanci A. (2005). Adsorption and kinetic studies of cationic and anionic dyes on pyrophyllite from aqueous solutions. J. Coll. Interf. Sci..

[37] Ho Y.S., McKay G. (1998). The kinetics of sorption of basic dyes from aqueous solution by sphagnum moss peat. Can. J. Chem. Eng..

[38] Mall I.D., Srivastava V.C., Agarwal N.K. (2006). Removal of Orange-G and Methyl Violet dyes by adsorption onto bagasse fly ash-kinetic study and equilibrium isotherm analyses. Dye. Pigment..

[39] Kuang Y., Zhang X., Zhou S. (2020). Adsorption of Methylene Blue in Water onto Activated Carbon by Surfactant Modification. Water.

